# Comparison of three non-insulin-based insulin resistance indexes in predicting the presence and severity of coronary artery disease

**DOI:** 10.3389/fcvm.2022.918359

**Published:** 2022-07-29

**Authors:** Zhenguo Wu, Huiliang Cui, Wei Li, Yerui Zhang, Li Liu, Zaibao Liu, Wencheng Zhang, Tengfei Zheng, Jianmin Yang

**Affiliations:** ^1^The Key Laboratory of Cardiovascular Remodeling and Function Research, Chinese Ministry of Education, Chinese National Health Commission and Chinese Academy of Medical Sciences, The State and Shandong Province Joint Key Laboratory of Translational Cardiovascular Medicine, Department of Cardiology, Qilu Hospital of Shandong University, Cheeloo College of Medicine, Shandong University, Jinan, China; ^2^Department of Cardiology, People’s Hospital of Qihe County, Dezhou, China

**Keywords:** coronary artery disease, triglyceride to high-density lipoprotein cholesterol (TG/HDL-C) ratio, triglyceride and glucose index (TyG index), metabolic score for insulin resistance (METS-IR), Gensini score

## Abstract

**Background:**

Insulin resistance (IR) has emerged as a risk factor for coronary heart disease (CAD), but there is currently insufficient data on the association of non-insulin-based IR indexes [triglyceride (TG)/high-density lipoprotein cholesterol (HDL-C) ratio, triglyceride and glucose (TyG) index, and metabolic score for IR (METS-IR)] with the presence and severity of CAD. Thus, the present study aimed to examine the relationship between these three non-insulin-based IR indexes and CAD, as well as to further compare the predictive values of each index.

**Materials and methods:**

In total, 802 consecutive patients who underwent coronary angiography for suspected CAD from January 2016 to April 2017 were included in this study and were divided into the control group (*n* = 149) and CAD group (*n* = 653) according to the angiography results. The triglyceride to high-density lipoprotein cholesterol (TG/HDL-C) ratio, triglyceride and glucose index (TyG index), and METS-IR were calculated according to the corresponding formulas. The severity of CAD was evaluated using the Gensini score (GS). The relationship of the TG/HDL-C ratio, TyG index, and METS-IR with CAD was analyzed, and the predictive values of the indexes were compared.

**Results:**

The TG/HDL-C ratio, TyG index, and METS-IR in the CAD group were significantly higher than those in the control group. The TG/HDL-C ratio and METS-IR in the high GS group were significantly higher than those in the non-high GS group. Multivariate logistic regression analysis showed that the TG/HDL-C ratio and METS-IR were independent predictors for the presence of CAD {adjusted odds ratio (OR) [95% confidence interval (CI)]: 1.32 (1.02–1.70) and 1.65 (1.32–2.05), respectively}, whereas only the METS-IR was an independent predictor of the severity of CAD [adjusted OR (95% CI): 1.22 (1.02–1.47)]. Further subgroup analysis indicated that statistical significance was observed only among men, younger patients (≤ 60), and patients with prediabetes mellitus (PDM). Receiver operator characteristic (ROC) analysis showed that the METS-IR had the highest predictive value for the prediction of both the presence and severity of CAD.

**Conclusion:**

The TG/HDL-C ratio, TyG index, and METS-IR are valuable predictors of the presence and severity of CAD, and the METS-IR has the highest predictive value among the three non-insulin-based IR indexes.

## Introduction

Despite ongoing advances in the prevention, diagnosis, and treatment of atherosclerosis, coronary artery disease (CAD) remains one of the leading causes of death, disability, and high healthcare costs worldwide (1, 2). Therefore, it is crucial to identify novel predictors for CAD. There is a growing body of evidence showing that insulin resistance (IR), which is a prominent characteristic of the metabolic syndrome and type 2 diabetes mellitus (T2DM), may also be involved in the pathogenesis of CAD (3–5). The gold standard for the assessment of insulin action *in vivo* is the hyperinsulinemic–euglycemic clamp technique (6), but its clinical use is limited due to experimental complexity and high cost (7). The most widely used method for the evaluation of insulin sensitivity is homeostasis model assessment for IR (HOMA-IR) (8), which is easily affected by the limited precision of insulin measurements (7). In this regard, some non-insulin-based IR indexes, such as the triglyceride to high-density lipoprotein cholesterol (TG/HDL-C) ratio, triglyceride and glucose index (TyG index), and the metabolic score for IR (METS-IR), have been evaluated as surrogates for IR (9–11). These novel indicators are calculated using simple routine biochemical tests, and they compensate for the shortcomings of traditional IR assessment methods. Previous studies have shown that non-insulin-based IR indexes are associated with multiple risk factors of cardiovascular disease (CVD), such as hypertension, diabetes, obesity, and metabolic syndrome, and they also predict the incidence and prognosis of CVD (12–16). To our knowledge, no research has specifically focused on the relationship between these three non-insulin-based IR indexes and the severity of CAD. Therefore, we aimed to investigate the value of the TG/HDL-C ratio, TyG index, and METS-IR in predicting the risk and severity of CAD in this cross-sectional study.

## Materials and methods

### Study population

The present study complied with the Declaration of Helsinki and was approved by the Ethics Review Committee of Qilu Hospital of Shandong University. All patients provided informed consent.

This was an observational study involving patients with known or suspected CAD who underwent coronary angiography at Qilu Hospital of Shandong University between January 2016 and April 2017. A total of 1,137 consecutive patients were examined. The exclusion criteria were as follows: history of previous coronary intervention or coronary artery bypass graft, severe valvular heart disease, decompensated heart failure, non-ischemic dilated cardiomyopathy, cerebrovascular disease, symptomatic peripheral arterial disease, renal or hepatic disease (serum creatinine > 1.4 mg/dl or liver function parameters > 3 times the upper normal value), acute or chronic infection and/or inflammation, chronic obstructive lung disease, thyroid and adrenal cortex dysfunction, malignancy, hematologic disease, autoimmune disease, or incomplete medical records. Finally, a total of 802 patients were enrolled in the present study. According to our pre-experimental results, we selected the research factor with the smallest OR (the TyG index) to calculate the sample size. Considering α = 0.05, β = 0.20, and OR = 1.30, a sample size of 603 was required. The sample size of the present study exceeded the calculated sample size.

### Data collection

Clinical data, including patients’ demographic data, medical history, laboratory tests, and basic cardiovascular medication information, were collected from medical records by trained clinicians. Resting systolic blood pressure (SBP) and diastolic blood pressure (DBP) were measured three times on the right arm and the mean value was considered as the participant’s blood pressure. All blood samples of subjects were collected in the morning after overnight fasting (8 h minimum). The levels of fasting plasma glucose (FPG), glycated hemoglobin (HbA1c), total cholesterol (TC), triglyceride (TG), low-density lipoprotein cholesterol (LDL-C), high-density lipoprotein cholesterol (HDL-C), serum creatinine (SCr), and uric acid (UA) were measured using an automatic biochemical analyzer.

### Definition of terms

Body mass index (BMI) was calculated as weight (kg) divided by the square of height (m^2^). Family history of CAD (FH-CAD) was defined as a history of CAD in a first-degree relative < 55 years (male) or <65 years (female). Hypertension was defined as either repeated blood pressure measurements of a SBP≥140 mmHg and/or a DBP≥90 mmHg at rest or the use of antihypertensive medication. According to the American Diabetes Association’s Standards of Medical Care in Diabetes (17), diabetes mellitus (DM) was defined as FPG≥7.0 mmol/L, random blood glucose (RBG)≥11.1 mmol/L, 2 h plasma glucose after oral glucose tolerance test (OGTT)≥11.1 mmol/L, plasma HbA1c of ≥ 6.5%, or the use of insulin or oral hypoglycemic agents, and prediabetes mellitus (PDM) was defined as 5.6 mmol/L≤FPG < 7.0 mmol/L, 7.8 mmol/L≤2 h plasma glucose < 11.1 mmol/L, or plasma HbA1c of ≥ 5.7% but <6.5%. The estimated glomerular filtration rate (eGFR) was calculated using SCr according to the Chinese modified Modification of Diet in Renal Disease equation as follows (18): *eGFR*(*mL*/*min*/1.73*m*^2^) = 175×*SCr*(*mg*/*dL*)^−1.234^×age(year)^−0.179^×0.79(*iffemale*). Non-insulin-based IR indexes were calculated by the following formulas: TyG=*Ln*[*fastingTG*(*mg*/*dL*)×*FPG*(*mg*/*dL*)÷2] (19); *TG*/*HDL*−C=*TG*(*mg*/*dL*)÷HDL−C(*mg*/*dL*) (10); and METS−IR=*ln*[(2×*FPG*(*mg*/*dL*)) + *fastingTG*(*mg*/*dL*)]×*BMI*(*kg*/*m*^2^)÷Ln[HDL−C(*mg*/*dL*)] (11).

### Coronary angiography

Standard Judkins technique was used for coronary angiography. At least two different plane images were taken for each coronary artery. The results were analyzed by at least two experienced interventional cardiologists. CAD was defined as the presence of obstructive stenosis of >50% of the vessel lumen diameter in any of the main coronary arteries, including the left main coronary artery (LM), left anterior descending artery (LAD), left circumflex coronary artery (LCX), and right coronary artery (RCA), or main branches of the vascular system. The severity of CAD was evaluated by the Gensini score (GS) (20) according to the following scale: 1 point, <25% narrowing; 2 points, 26–50% narrowing; 4 points, 51–75% narrowing; 8 points, 76–90% narrowing; 16 points, 91–99% narrowing; and 32 points, total occlusion. Each segment was followed by a multiplying factor depending on the functional significance of the area supplied by that segment as follows: 5 for LM; 2.5 for the proximal segment of LAD and LCX; 1.5 for the middle segment of LAD; 1 for the distal segment of LAD, LCX, first diagonal branch, first obtuse marginal branch, RCA, and posterior descending artery; and 0.5 for other segments. The patients with angiographically defined CAD were divided into three groups based on the tertile of the GS as follows: low GS, <32 points (*n* = 221); intermediate GS, 32–64 points (*n* = 215); and high GS, 32–64 points (*n* = 217).

### Statistical analysis

Statistical analysis was performed using SPSS version 25.0 (SPSS, Chicago, IL, United States) and R software version 4.1.3 (R Foundation for Statistical Computing, Vienna, Austria). Receiver operator characteristic (ROC) curves were generated by MedCalc software version 19.6.4 (MedCalc Software Ltd., Ostend, Belgium). PASS version 15.0.1 (NCSS Statistical Software) was used for the sample size calculation. Continuous variables with a normal distribution are presented as the mean ± standard deviation (SD), and continuous variables with non-normal distribution are presented as the median with the 25th and 75th percentiles. Categorical variables are presented as the number and percentage. Independent samples *t*-test or analysis of variance (ANOVA) was used to compare continuous variables with a normal distribution. For continuous variables with non-normal distribution, Mann–Whitney *U* test or Kruskal–Wallis *H* test was used. Categorical variables were analyzed using the chi-squared test or Fisher’s exact test. The association between GS and non-insulin-based IR indexes was assessed using Spearman’s correlation analysis. Variables were analyzed by univariate logistic regression analysis, and variables < 0.1 in univariate analysis were included in the multivariate logistic regression analysis. The variance inflation factor (VIF) of the variables included in the models was calculated to avoid the result deviation caused by multicollinearity. No evidence of collinearity was found in the models given a VIF≥1.32. We standardized the TG/HDL-C ratio, TyG index, and METS-IR using regression analysis to determine the relationships between the increase of non-insulin-based IR indexes per SD and the presence and severity of CAD. We also performed a subgroup analysis based on gender, age, and DM state to determine the association between the METS-IR and the severity of CAD differed across various subgroups, and *p* for interaction was calculated. ROC curves were generated for the TG/HDL-C ratio, TyG index, and METS-IR. The maximum Youden index was used to determine the optimal cutoff value. The area under the curve (AUC) was used to compare the diagnostic utility of the presence of CAD and a high GS. A *p*-value of less than 0.05 was considered statistically significant.

## Results

### Baseline characteristics

In total, 802 patients were enrolled in the present study, including 653 patients with angiographically defined CAD (CAD group) and 149 patients with normal coronary angiography (control group). The baseline clinical and demographic characteristics are shown in Table 1. A comparison between the CAD group and the control group revealed no significant difference in BMI, SBP, DBP, LVEF, smoking status, FH-CAD, glucose metabolism status, TC, TG, and LDL-C. However, age, FPG, and UA were higher in the CAD group than in the control group, whereas the HDL-C and eGFR were lower among subjects with CAD. In addition, the percentages of men, hypertension, and cardiovascular medication use were higher in the CAD group compared to the control group. Moreover, the CAD group presented a significantly higher TG/HDL-C ratio, TyG index, and METS-IR compared to the control group [2.66 (1.90–3.74) vs. 2.41 (1.67–3.23), *p* = 0.010; 8.63 (8.31–8.99) vs. 8.47 (8.16–8.90), *p* = 0.019; 40.06 (35.50–44.13) vs. 36.91 (33.35–41.20), *p* < 0.001, respectively].

**TABLE 1:** Baseline characteristics of the study population.

Variables	Total (*n* = 802)	Control group (*n* = 149)	CAD group (*n* = 653)	*p*-value
**General conditions**				
Age (years)	58.94 ± 10.18	53.93 ± 11.28	60.08 ± 9.56	**<0.001**
Male, *n* (%)	534 (66.6)	83 (55.7)	451 (69.1)	**0.002**
BMI (kg/m^2^)	26.04 ± 3.26	25.75 ± 3.54	26.10 ± 3.20	0.236
SBP (mmHg)	133.53 ± 17.84	132.16 ± 15.63	133.85 ± 18.30	0.252
DBP (mmHg)	75.79 ± 11.15	76.17 ± 10.50	75.70 ± 11.30	0.643
LVEF (%)	59.66 ± 10.07	60.52 ± 8.45	59.46 ± 10.40	0.087
**Risk factors, *n* (%)**				
Current smoking	80 (10.0)	12 (8.1)	68 (10.4)	0.386
FH-CAD	234 (29.2)	37 (24.8)	197 (30.2)	0.196
Glucose metabolism status				**<0.001**
NGR	484 (60.3)	116 (77.9)	368 (56.4)	
PDM	105 (13.1)	12 (8.1)	93 (14.2)	
DM	213 (26.6)	21 (14.1)	192 (29.4)	
Hypertension	494 (61.6)	80 (53.7)	414 (63.4)	**0.028**
**Laboratory test**				
FPG (mg/dL)	91.72 (83.25–108.48)	86.50 (79.38–97.40)	92.98 (84.42–111.00)	**<0.001**
TC (mg/dL)	148.45 (127.58–174.74)	155.03 (132.80–176.87)	146.91 (126.61–174.55)	0.128
TG (mg/dL)	115.11 (88.55–154.07)	110.68 (80.58–154.95)	115.99 (89.43–154.07)	0.341
LDL-C (mg/dL)	88.53 (71.52–110.95)	91.62 (71.52–108.63)	87.76 (71.52–111.34)	0.831
HDL-C (mg/dL)	44.46 (39.05–51.80)	47.55 (42.53–55.09)	43.69 (38.27–51.03)	**<0.001**
eGFR (mL/min/1.73 m^2^)	104.36 (92.03–119.80)	108.10 (98.86–124.47)	103.02 (91.34–117.36)	**0.001**
UA (μmol/L)	303.00 (257.00–357.25)	294.00 (228.00–347.00)	304.00 (263.50–358.50)	**0.011**
**Cardiovascular medications, *n* (%)**				
Single antiplatelet therapy	152 (19.0)	14 (9.4)	138 (21.1)	**<0.001**
Dual antiplatelet therapy	20 (2.5)	1 (0.7)	19 (2.9)	0.114
Beta-blocker	52 (6.5)	9 (6.0)	43 (6.6)	0.807
ACEI/ARB	73 (9.1)	15 (10.1)	58 (8.9)	0.650
Statin	135 (16.8)	16 (10.7)	119 (18.2)	**0.028**
**Diabetic medications, *n* (%)**				
Insulin	109 (13.6)	11 (7.4)	98 (15.0)	**0.014**
Metformin	76 (9.5)	9 (6.0)	67 (10.3)	0.113
Other hypoglycemic drugs	110 (13.7)	5 (3.4)	105 (16.1)	**<0.001**
TG/HDL-C ratio	2.59 (1.86–3.66)	2.41 (1.67–3.23)	2.66 (1.90–3.74)	**0.010**
TyG index	8.62 (8.29–8.98)	8.47 (8.16–8.90)	8.63 (8.31–8.99)	**0.019**
METS-IR	39.35 (35.00–43.64)	36.91 (33.35–41.20)	40.06 (35.50–44.13)	**<0.001**

Data were given as mean ± SD, median with interquartile range or n (%).

p-values in bold are <0.05.

BMI, body mass index; SBP, systolic blood pressure; DBP, diastolic blood pressure; LVEF, left ventricular ejection fraction; FH-CAD, family history of coronary artery disease; NGR, normal glucose regulation; PDM, prediabetes mellitus; DM, diabetes mellitus; FPG, fasting plasma glucose; TC, total cholesterol; TG, triglyceride; LDL-C, low-density lipoprotein-cholesterol; HDL-C, high-density lipoprotein-cholesterol; eGFR, estimated glomerular filtration rate; UA, uric acid; ACEI, angiotensin-converting enzyme inhibitors; ARB, angiotensin receptor blockers; TG/HDL-C ratio, the ratio of triglycerides to high-density lipoprotein cholesterol; TyG index, triglyceride and glucose index; METS-IR, metabolic score for insulin resistance.

**FIGURE 1:**
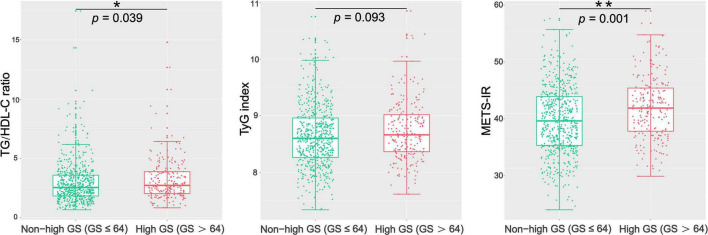
Comparison of the TG/HDL-C ratio, TyG index, and METS-IR between the high GS group and the non-high GS group. GS, Gensini score; TG/HDL-C ratio, the ratio of triglycerides to high-density lipoprotein cholesterol; TyG index, triglyceride and glucose index; METS-IR, metabolic score for insulin resistance. *p < 0.05; **p < 0.01.

The patients with CAD were categorized into three groups according to their GS as follows: low GS, <32 points (*n* = 221); intermediate GS, 32–64 points (*n* = 215); and high GS, 32–64 points (*n* = 217). The baseline characteristics according to the tertile of the GS are summarized in Table 2. As shown in Table 2, there were significant differences in gender, LVEF, glucose metabolism status, HDL-C, FH-CAD frequency, and METS-IR. However, no statistical difference was observed in the TG/HDL-C ratio and TyG index among the groups based on the tertile of GS (*p* = 0.115 and 0.119, respectively). More importantly, the TG/HDL-C ratio and METS-IR were significantly different between the high GS group and non-high GS group (*p* = 0.039 and 0.001, respectively), whereas the TyG index showed no statistical difference (*p* = 0.093) (Figure 1).

**TABLE 2:** Baseline characteristics of the study population according to the tertile of the Gensini score.

Variables	Low GS (GS < 32, *n* = 221)	Intermediate GS (GS: 32–64, *n* = 215)	High GS (GS > 64, *n* = 217)	*p*-value
**General conditions**				
Age (years)	60.22 ± 9.77	59.63 ± 8.97	60.39 ± 9.92	0.684
Male, *n* (%)	133 (60.2)	158 (73.5)	160 (73.7)	**0.002**
BMI (kg/m^2^)	25.84 ± 3.39	26.02 ± 3.24	26.45 ± 2.92	0.124
SBP (mmHg)	134.59 ± 17.06	132.48 ± 17.80	134.44 ± 19.96	0.408
DBP (mmHg)	76.39 ± 11.52	75.30 ± 11.42	75.38 ± 10.97	0.530
LVEF (%)	61.08 ± 10.02	58.34 ± 10.69	58.92 ± 10.34	**0.014**
**Risk factors, *n* (%)**				
Current smoking	18 (8.1)	29 (13.5)	21 (9.7)	0.172
FH-CAD	38 (17.2)	56 (26.0)	103 (47.5)	**<0.001**
Glucose metabolism status				**0.018**
NGR	137 (62.0)	127 (59.1)	104 (47.9)	
PDM	32 (14.5)	29 (13.5)	32 (14.7)	
DM	52 (23.5)	59 (27.4)	81 (37.3)	
Hypertension	150 (67.9)	130 (60.5)	134 (61.8)	0.228
**Laboratory test**				
FPG (mg/dl)	92.26 (83.70–109.74)	91.00 (84.15–108.48)	94.60 (85.14–114.70)	0.104
TC (mg/dl)	149.61 (129.12–175.71)	141.88 (127.19–164.31)	148.84 (123.13–178.80)	0.114
TG (mg/dl)	120.42 (88.55–157.17)	114.22 (88.55–146.10)	115.99 (91.64–157.17)	0.396
LDL-C (mg/dl)	87.73 (70.75–113.66)	85.44 (72.29–103.61)	92.40 (71.33–116.37)	0.103
HDL-C (mg/dl)	46.39 (40.98–52.96)	43.30 (37.89–51.42)	42.14 (37.50–48.33)	**<0.001**
eGFR (ml/min/1.73 m^2^)	103.42 (93.14–117.45)	103.00 (89.56–116.38)	103.52 (89.22–119.54)	0.770
UA (μmol/L)	303.00 (258.50–352.00)	306.00 (262.00–368.00)	304.00 (270.00–355.50)	0.301
**Cardiovascular medications, *n* (%)**				
Single antiplatelet therapy	41 (18.6)	54 (25.1)	43 (19.8)	0.206
Dual antiplatelet therapy	0	7 (3.3)	12 (5.5)	**0.002**
Beta-blocker	16 (7.2)	15 (7.0)	12 (5.5)	0.741
ACEI/ARB	18 (8.1)	22 (10.2)	18 (8.3)	0.696
Statin	37 (16.7)	48 (22.3)	34 (15.7)	0.157
**Diabetic medications, *n* (%)**				
Insulin	31 (14.0)	25 (11.6)	42 (19.4)	0.070
Metformin	24 (10.9)	21 (9.8)	22 (10.1)	0.929
Other hypoglycemic drugs	29 (13.1)	31 (14.4)	45 (20.7)	0.069
TG/HDL-C ratio	2.48 (1.83–3.66)	2.73 (1.86–3.63)	2.71 (2.02–3.88)	0.115
TyG index	8.63 (8.27–9.00)	8.58 (8.30–8.89)	8.66 (8.36–9.03)	0.119
METS-IR	38.80 (34.18–43.70)	39.18 (35.00–44.12)	41.01 (36.71–44.75)	**0.005**

Data were given as mean ± SD, median with interquartile range or n (%).

p-values in bold are < 0.05.

BMI, body mass index; SBP, systolic blood pressure; DBP, diastolic blood pressure; LVEF, left ventricular ejection fraction; FH-CAD, family history of coronary artery disease; NGR, normal glucose regulation; PDM, prediabetes mellitus; DM, diabetes mellitus; FPG, fasting plasma glucose; TC, total cholesterol; TG, triglyceride; LDL-C, low-density lipoprotein-cholesterol; HDL-C, high-density lipoprotein-cholesterol; eGFR, estimated glomerular filtration rate; UA, uric acid; ACEI, angiotensin-converting enzyme inhibitors; ARB, angiotensin receptor blockers; TG/HDL-C ratio, the ratio of triglycerides to high-density lipoprotein cholesterol; TyG index, triglyceride and glucose index; METS-IR, metabolic score for insulin resistance.

In addition, the baseline characteristics were described according to the glucose metabolism status. As shown in Supplementary Table 1, patients with PDM and DM had higher percentages of CAD and higher GS, TG/HDL-C ratio, TyG index, and METS-IR (Supplementary Table 1). The patients with DM were further grouped by metformin use. The proportion of CAD was similar in the two groups, whereas patients with metformin had a lower GS. Moreover, the differences in the TG/HDL-C ratio, TyG index, and METS-IR between the two groups were not statistically significant (Supplementary Table 2).

### Correlations among triglyceride to high-density lipoprotein cholesterol ratio, triglyceride and glucose index, metabolic score for insulin resistance, and Gensini score

**FIGURE 2:**
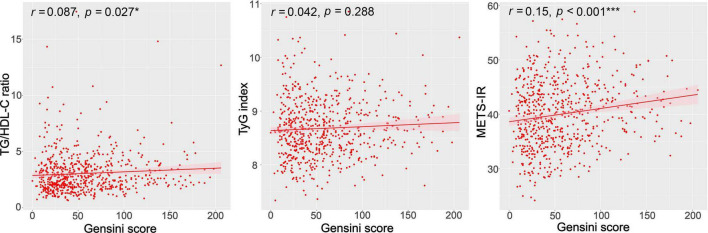
Correlations between the TG/HDL-C ratio, TyG index, METS-IR, and the GS. GS, Gensini score; TG/HDL-C ratio, the ratio of triglycerides to high-density lipoprotein cholesterol; TyG index, triglyceride and glucose index; METS-IR, metabolic score for insulin resistance. *p < 0.05; ***p < 0.001.

We used Spearman’s correlation analyses to examine the correlations of the TG/HDL-C ratio, TyG index, and METS-IR with GS in patients with CAD. As shown in Figure 2, the GS was significantly positively correlated with the TG/HDL-C ratio and METS-IR (*r* = 0.087, *p* = 0.027; *r* = 0.151, *p* < 0.001, respectively). No significant correlation was observed between the TyG index and GS (*r* = 0.042, *p* = 0.288) (Figure 2).

### Univariate and multivariate analyses

Univariate analysis demonstrated that the following factors were associated with the presence of CAD: age; gender; glucose metabolism status; hypertension; eGFR; UA; single antiplatelet therapy; use of statin, insulin, and other hypoglycemic drugs; TG/HDL-C ratio; TyG index; and METS-IR (Table 3). Multivariate logistic regression analysis showed that the TG/HDL-C ratio and METS-IR remained significant after adjusting for confounders. For each SD increase in the TG/HDL-C ratio and METS-IR, the adjusted OR (95% CI) was 1.32 (1.02–1.70) and 1.65 (1.32–2.05), respectively. In addition, age, male, glucose metabolism status, UA, single antiplatelet therapy, and the use of hypoglycemic drugs were independently associated with the present of CAD. However, the TyG index was not an independent predictor for CAD (Table 4).

**TABLE 3:** Univariate logistic regression analyses for the presence of CAD.

Variables	OR	95% CI	*p*-value
Age (years)	1.06	1.04–1.08	**<0.001**
Male	1.77	1.24–2.56	**0.002**
SBP (mmHg)	1.01	1.00–1.02	0.298
DBP (mmHg)	1.00	0.98–1.01	0.643
LVEF (%)	0.33	0.05–2.12	0.245
Current smoking	1.33	0.70–2.52	0.387
FH-CAD	1.31	0.87–1.97	0.197
Glucose metabolism status			
NGR	1 (reference)		
PDM	2.44	1.29–4.62	**0.006**
DM	2.88	1.75–4.74	**<0.001**
Hypertension	1.49	1.04–2.14	**0.028**
TC (mg/dL)	1.00	0.99–1.00	0.336
LDL-C (mg/dL)	1.00	1.00–1.01	0.690
eGFR (mL/min/1.73 m^2^)	0.99	0.98–1.00	**0.001**
UA (μmol/L)	1.00	1.00–1.01	**0.007**
Single antiplatelet therapy	2.58	1.45–4.62	**0.001**
Dual antiplatelet therapy	4.44	0.59–33.40	0.148
Beta-blocker	1.10	0.52–2.30	0.808
ACEI/ARB	0.87	0.48–1.58	0.650
Statin	1.85	1.06–3.23	**0.030**
Insulin	2.22	1.16–4.25	**0.017**
Metformin	1.78	0.87–3.65	0.117
Other hypoglycemic drugs	5.52	2.21–13.79	**<0.001**
TG/HDL-C ratio (Per SD)	1.32	1.06–1.65	**0.015**
TyG index (Per SD)	1.24	1.03–1.50	**0.024**
METS-IR (Per SD)	1.73	1.42–2.10	**<0.001**

p-values in bold are <0.05.

OR, odds ratio; CI, confidence interval; SBP, systolic blood pressure; DBP, diastolic blood pressure; LVEF, left ventricular ejection fraction; FH-CAD, family history of coronary artery disease; NGR, normal glucose regulation; PDM, prediabetes mellitus; DM, diabetes mellitus; FPG, fasting plasma glucose; TC, total cholesterol; TG, triglyceride; LDL-C, low-density lipoprotein-cholesterol; HDL-C, high-density lipoprotein-cholesterol; eGFR, estimated glomerular filtration rate; UA, uric acid; ACEI, angiotensin-converting enzyme inhibitors; ARB, angiotensin receptor blockers; TG/HDL-C ratio, the ratio of triglycerides to high-density lipoprotein cholesterol; TyG index, triglyceride and glucose index; METS-IR, metabolic score for insulin resistance.

**TABLE 4:** Multivariate logistic regression analyses for the presence of CAD.

Variables	Model 1		*p*-value	Model 2		*p*-value	Model 3		*p*-value
	OR	95% CI		OR	95% CI		OR	95% CI	
Age (years)	1.08	1.05–1.10	**<0.001**	1.08	1.05–1.10	**<0.001**	1.08	1.06–1.10	**<0.001**
Male, *n* (%)	2.24	1.45–3.47	**<0.001**	2.30	1.48–3.58	**<0.001**	2.10	1.35–3.26	**0.001**
Glucose metabolism status									
NGR	Reference			Reference			Reference		
PDM	2.02	1.03–3.94	**0.040**	2.04	1.03–4.03	**0.041**	1.74	0.88–3.43	0.113
DM	1.89	0.84–4.23	0.123	1.81	0.80–4.10	0.158	1.94	0.85–4.43	0.118
Hypertension	1.17	0.78–1.74	0.445	1.18	0.79–1.77	0.406	1.04	0.69–1.57	0.859
eGFR (mL/min/1.73 m^2^)	1.00	0.99–1.01	0.818	1.00	0.99–1.01	0.820	1.00	0.99–1.01	0.764
UA (μmol/L)	1.00	1.00–1.01	**0.023**	1.00	1.00–1.01	**0.015**	1.00	1.00–1.01	0.065
Single antiplatelet therapy	2.89	1.25–6.71	**0.013**	2.90	1.25–6.70	**0.013**	2.94	1.26–6.88	**0.013**
Statin	0.84	0.37–1.90	0.673	0.82	0.36–1.84	0.624	0.86	0.37–1.98	0.723
Insulin	1.03	0.39–2.75	0.948	1.02	0.39–2.71	0.966	0.86	0.31–2.35	0.766
Other hypoglycemic drugs	3.09	1.05–9.10	**0.041**	3.07	1.04–9.06	**0.042**	2.64	0.88–7.92	0.082
TG/HDL-C ratio (Per SD)	1.32	1.02–1.70	**0.033**	–			–		
TyG index (Per SD)	–			1.28	0.84–1.95	0.256	–		
METS-IR (Per SD)	–			–			1.65	1.32–2.05	**<0.001**

p-values in bold are <0.05.

OR, odds ratio; CI, confidence interval; NGR, normal glucose regulation; PDM, prediabetes mellitus; DM, diabetes mellitus; eGFR, estimated glomerular filtration rate; UA, uric acid; TG/HDL-C ratio, the ratio of triglycerides to high-density lipoprotein cholesterol; TyG index, triglyceride and glucose index; METS-IR, metabolic score for insulin resistance.

The results of multivariate logistic regression analysis for predicting high GS suggested that only the METS-IR was an independent predictor of the severity of CAD (Table 5). For each SD increase in the METS-IR, the adjusted OR (95% CI) was 1.22 (1.02–1.47). Furthermore, FH-CAD, LDL-C, and dual antiplatelet therapy were independent predictors of high GS (Table 5).

**TABLE 5:** Univariate and multivariate logistic regression analyses for high GS.

Variables	Univariate		*p*-value	Multivariate		*p*-value
	OR	95% CI		OR	95% CI	
Age (years)	1.01	0.99–1.02	0.560			
Male, *n* (%)	1.40	0.97–2.01	0.069	1.39	0.94–2.06	0.097
LVEF (%)	0.48	0.10–2.23	0.347			
SBP (mmHg)	1.00	0.99–1.01	0.559			
DBP (mmHg)	1.00	0.98–1.01	0.614			
Current smoking	0.89	0.52–1.53	0.664			
FH-CAD	3.29	2.32–4.67	**<0.001**	3.21	2.24–4.61	**<0.001**
Glucose metabolism status						
NGR	Reference			Reference		
PDM	1.33	0.82–2.16	0.246	1.16	0.68–1.97	0.583
DM	1.85	1.29–2.67	**0.001**	1.65	0.89–3.04	0.113
Hypertension	0.90	0.64–1.26	0.537			
TC (mg/dL)	1.00	1.00–1.01	0.356			
LDL-C (mg/dL)	1.01	1.00–1.01	**0.028**	1.01	1.00–1.01	**0.031**
eGFR (mL/min/1.73 m^2^)	1.00	0.99–1.01	0.941			
UA (μmol/L)	1.00	1.00–1.00	0.500			
Single antiplatelet therapy	0.89	0.59–1.33	0.561			
Dual antiplatelet therapy	3.59	1.39–9.25	**0.008**	3.77	1.37–10.40	**0.010**
Beta-blocker	0.77	0.39–1.52	0.444			
ACEI/ARB	0.90	0.50–1.60	0.710			
Statin	0.77	0.50–1.19	0.234			
Insulin	1.63	1.05–2.52	**0.029**	1.21	0.66–2.24	0.537
Metformin	0.98	0.57–1.68	0.942			
Other hypoglycemic drugs	1.64	1.07–2.51	**0.023**	0.89	0.48–1.64	0.703
TG/HDL-C ratio	1.13	0.97–1.32	0.113			
TyG index	1.15	0.98–1.35	0.091			
METS-IR	1.32	1.12–1.55	**0.001**	1.22	1.02–1.47	**0.032**

p-values in bold are <0.05.

OR, odds ratio; CI, confidence interval; SBP, systolic blood pressure; DBP, diastolic blood pressure; LVEF, left ventricular ejection fraction; FH-CAD, family history of coronary artery disease; NGR, normal glucose regulation; PDM, prediabetes mellitus; DM, diabetes mellitus; FPG, fasting plasma glucose; TC, total cholesterol; TG, triglyceride; LDL-C, low-density lipoprotein-cholesterol; HDL-C, high-density lipoprotein-cholesterol; eGFR, estimated glomerular filtration rate; UA, uric acid; ACEI, angiotensin-converting enzyme inhibitors; ARB, angiotensin receptor blockers; TG/HDL-C ratio, the ratio of triglycerides to high-density lipoprotein cholesterol; TyG index, triglyceride and glucose index; METS-IR, metabolic score for insulin resistance.

### Subgroup analyses

**FIGURE 3:**
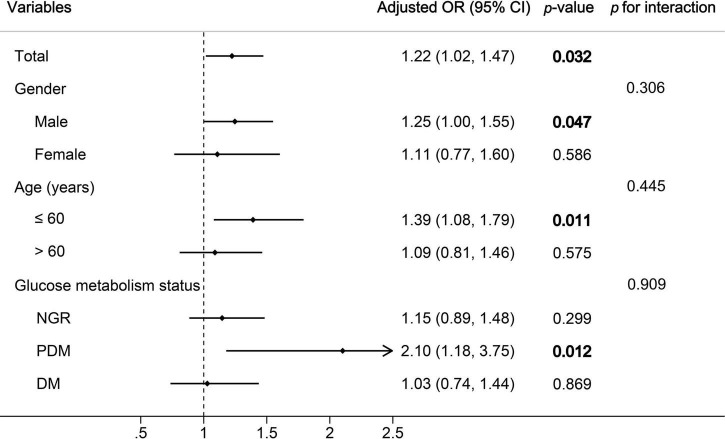
Subgroup and interaction analyses between the METS-IR (Per SD) and high GS across various subgroups. OR, odds ratio; CI, confidence interval; GS, Gensini score; NGR, normal glucose regulation; PDM, prediabetes mellitus; DM, diabetes mellitus. *p*-values in bold are <0.05.

The association between the METS-IR and the severity of CAD was examined by subgroup analyses. Although no interaction was found between age, gender, glucose metabolism status, and METS-IR for the high GS in multivariate analysis (all *p*-values for interaction≥0.306), the statistical significance was observed only among men, the younger age group (≤ 60), and patients with PDM (Figure 3).

### Receiver operator characteristic curve analysis

**FIGURE 4:**
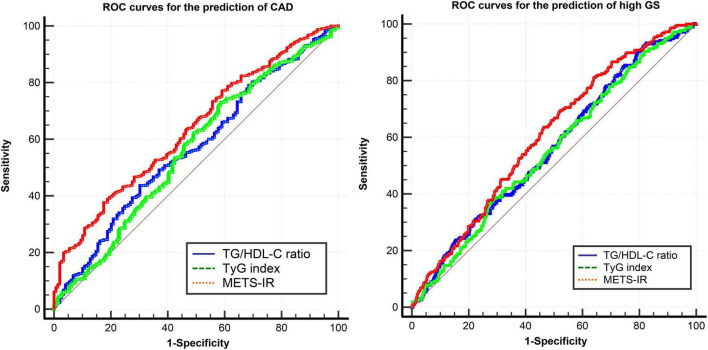
ROC curves of the TG/HDL-C ratio, TyG index, and METS-IR for the prediction of CAD and high GS. ROC curves, receiver operator characteristic curves; CAD, coronary heart disease; GS, Gensini score; TG/HDL-C ratio, the ratio of triglycerides to high-density lipoprotein cholesterol; TyG index, triglyceride and glucose index; METS-IR, metabolic score for insulin resistance.

The ROC analysis demonstrated that the optimal cutoff values of the TG/HDL-C ratio, TyG index, and METS-IR for predicting the presence of CAD were 2.9, 8.3, and 42.1 respectively. While comparing the predictive power, the METS-IR [AUC (95%CI): 0.636 (0.589–0.683)] demonstrated the highest AUC value compared to the TG/HDL-C ratio [0.567 (0.517–0.618)] and TyG index [0.562 (0.509–0.614)] (Figure 4 and Table 6). For the prediction of high GS (GS > 64), the METS-IR had the highest AUC at 0.606 (95% CI: 0.564–0.648, *p* < 0.001) among the non-insulin-based IR indexes. In addition, the METS-IR of 38.2 was identified as the optimal cutoff point for detecting high GS with a sensitivity of 69.1% and a specificity of 48.2% (Figure 4 and Table 7).

**TABLE 6:** Comparison of the predictive value of the TG/HDL-C ratio, TyG index, and METS-IR for predicting the presence of CAD.

Variables	Sensitivity (%)	Specificity (%)	Cutoff value	AUC (95% CI)	*p*-value	Comparison of AUC	
						Difference	*p*-value
TG/HDL-C ratio	43.6	69.8	>2.9	0.567 (0.517–0.618)	**0.009**	–0.069	**0.015**
TyG index	73.0	41.6	>8.3	0.562 (0.509–0.614)	**0.022**	–0.074	**0.016**
METS-IR	37.7	82.6	>42.1	0.636 (0.589–0.683)	**<0.001**	Reference	

p-values in bold are <0.05.

CAD, coronary heart disease; TG/HDL-C ratio, the ratio of triglycerides to high-density lipoprotein cholesterol; TyG index, triglyceride and glucose index; METS-IR, metabolic score for insulin resistance; AUC, area under the curve; CI, confidence interval.

**TABLE 7:** Comparison of the predictive value of the TG/HDL-C ratio, TyG index, and METS-IR for predicting high GS.

Variables	Sensitivity (%)	Specificity (%)	Cutoff value	AUC (95% CI)	*p*-value	Comparison of AUC	
						Difference	*p*-value
TG/HDL-C ratio	93.1	17.9	>1.6	0.563 (0.519–0.607)	**0.006**	–0.043	**0.042**
TyG index	38.2	71.5	>8.9	0.553 (0.509–0.597)	**0.021**	–0.053	**0.022**
METS-IR	69.1	48.2	>38.2	0.606 (0.564–0.648)	**<0.001**	Reference	

p-values in bold are <0.05.

GS, Gensini score; TG/HDL-C ratio, the ratio of triglycerides to high-density lipoprotein cholesterol; TyG index, triglyceride and glucose index; METS-IR, metabolic score for insulin resistance; AUC, area under the curve; CI, confidence interval.

## Discussion

Our current study demonstrated that the TG/HDL-C ratio and METS-IR were independent predictors of the presence of CAD, and only the METS-IR was an independent predictor of high GS. More importantly, the METS-IR had the highest predictive value for the prediction of both the presence and severity of CAD.

Insulin resistance is a general term that characterizes a low response of adipose tissue, skeletal muscle, liver, and pancreas to insulin action. Theoretically, IR plays an important role in the pathogenesis of atherosclerosis, which is the most common cause of CAD. Several mechanisms describing the promotion of CAD by IR have been elucidated, including changes in classic CVD risk factors and alteration of insulin signaling pathways (21). The Insulin Resistance Atherosclerosis Study (IRAS) has revealed that higher levels of insulin sensitivity are associated with less atherosclerosis (22). A previous study has shown that IR is an important risk factor for CAD and is positively correlated with the severity of CAD, in which IR is measured using the hyperinsulinemic–euglycemic clamp technique (23). A 2012 meta-analysis of 65 studies has demonstrated that IR, as evaluated by HOMA-IR, is a good predictor of CVD (24). The hyperinsulinemic-euglycemic clamp technique is the gold standard to evaluate the degree of IR, but HOMA-IR is the most widely used method. However, the hyperinsulinemic–euglycemic clamp technique is costly and time-consuming (7), and HOMA-IR is likely to cause significant bias due to insulin measurements (25, 26). Recently, some non-insulin-based IR indexes, such as the TG/HDL-C ratio, TyG index, and METS-IR, have been used for the assessment of insulin action to compensate for the shortcomings of traditional IR assessment methods. Interestingly, these indexes have strong predictive abilities for the incidence and prognosis of CVD (12, 13, 15, 16, 27–29). To date, there has been no research to compare the value of the TG/HDL-C ratio, TyG index, and METS-IR in predicting the risk and severity of CAD.

In the present study, we found that the TG/HDL-C ratio, TyG index, and METS-IR were significantly higher in the CAD group than in the control group. The TG/HDL-C ratio and METS-IR were significantly different between the high GS group and non-high GS group, and both indexes were significantly positively correlated with GS. The proportion of current smoking was higher in the CAD group compared to the control group, but the difference was not statistically significant. Patients have different smoking characteristics, including years of smoking, the number of cigarettes, and secondhand-smoking exposure. All of these factors may affect CAD, which may have attributed to the lack of significance. The CAD group had a higher percentage of patients taking statins. The lack of significance in the TC, TG, and LDL-C between the groups may have been attributed to the higher usage of lipid-lowering drugs in the CAD group. There were more patients with hypertension in the CAD group, but the difference in SBP and DBP was not statistically significant between the groups. The use of anti-hypertensive drugs affects blood pressure, which may have attributed to the lack of significance. In the present study, patients with DM taking metformin had a lower GS. Previous studies have demonstrated that metformin improves cardiovascular functions and reduces cardiovascular risks (30, 31). In the present study, we found a potential connection between metformin and the severity of CAD.

Further analysis showed that the TG/HDL-C ratio and METS-IR were independent predictors of the presence of CAD, whereas only the METS-IR was an independent predictor of high GS. The correlations between GS and these indexes were relatively low, and the clinical application value of these indexes needs further research. In the present study, no statistical significance was found in the prediction of CAD severity by the TG/HDL-C ratio and TyG index after adjusting for confounders, which was consistent with previous studies (32, 33). Yunke et al. evaluated 317 consecutive patients who underwent coronary angiography and found that the TG/HDL-C ratio was predictive for CAD patients who had a GS greater than 40 even after adjusting for potential confounding variables (34). Mao et al. found that the TyG index is an independent predictor of the high SYNTAX score in an observational study that included 438 patients with non-ST-segment elevation acute coronary syndrome (35). The differences in the study population, tools for grading the severity of CAD, and the definition of severe CAD may have attributed to the inconsistent outcomes between the present study and previous studies.

In the present study, we found that single antiplatelet therapy was an independent predictive factor for the presence of CAD. However, dual antiplatelet therapy, instead of single antiplatelet therapy, was significantly associated with high GS. Single antiplatelet therapy is widely used for the prevention and treatment of CAD, whereas dual antiplatelet therapy is used for patients with acute coronary syndrome who may suffer from more severe CAD. The different indications of single and dual antiplatelet therapy may explain our findings.

In the subgroup analyses, the significant association between the METS-IR and high GS was mainly observed among men, younger patients, and patients with PDM. The Tehran Lipid and Glucose Study (TLGS) has also reported differences in the association between the TyG index and CVD in different age and gender groups (12). The inconsistency in the predictive power of non-insulin-based IR indexes between the age and gender groups needs further investigation to explore the underlying mechanism. IR occurs several years or even decades earlier than T2DM, which is an important risk factor for CVD in patients who develop T2DM (36). However, in patients with T2DM, the classic CVD risk factors are major predictors of CVD events, and the risk is further increased by hyperglycemia, but to a lesser extent as that by IR alone (37). These factors may explain the differences in the predictive power of the METS-IR between patients with different glucose metabolism statuses observed in the present study.

Prediabetes mellitus is an intermediate metabolic state between normal glucose regulation (NGR) and DM. Previous studies have shown that patients with PDM have a higher rate of CAD and a worse prognosis (30). Patients with PDM without obstructive coronary stenosis also have a worse prognosis caused by IR and endothelial dysfunction (30). In the present study, we observed a higher proportion of CAD and higher GS in patients with PDM, which was consistent with previous research. Further subgroup analysis showed that the METS-IR was significantly associated with high GS in patients with PDM, which has implications for severity stratification and early intervention of CAD in patients with PDM. Previous studies have linked the higher rate of acute myocardial infarction (AMI) in PDM with over-inflammation at the level of atherosclerotic plaques (38), peri-coronary fat (39, 40), and peripheral adipose tissue, as in the case of overweight (41). The ability of the METS-IR to reflect a hyperinflammatory state in patients with PDM is an important question to investigate. A previous prospective longitudinal observational study has reported that IR is a negative prognostic factor in subjects with ischemic heart disease and NGR (42). In the present study, the association between the METS-IR and high GS was not statistically significant in patients with NGR. Thus, the role of IR in CAD patients with NGR and the underlying mechanism need further investigation.

Bello-Chavolla et al. found that the METS-IR has a good and significantly higher diagnostic performance of incident T2DM compared to the TyG index and TG/HDL-C ratio in Mexican subjects (11). A study comparing the associations of the TG/HDL-C ratio, TyG index, and METS-IR with hypertension demonstrated that only the METS-IR is significantly associated with hypertension (43). In the present study, ROC analysis showed that the METS-IR had the highest predictive value for the prediction of both the presence and severity of CAD.

Several limitations of this study should be considered. First, this was a single-center retrospective study, indicating that potential bias may have been introduced. Second, as with many clinical studies, this cross-sectional study only showed association rather than causation. Third, the sample size was relatively small, which might influence our results. Fourth, the values of inflammatory markers were not measured in most patients, preventing the investigation of the associations between non-insulin-based IR indexes and inflammation. Finally, we did not record nutritional habits and physical activities, which may affect non-insulin-based IR indexes. Additional multicenter, large-size, and prospective studies may strengthen our conclusion.

## Conclusion

In conclusion, the present findings demonstrated that non-insulin-based IR indexes are valuable predictors of the presence and severity of CAD and that the METS-IR has the highest predictive value among the three non-insulin-based IR indexes. Thus, these findings suggested that the METS-IR is a simple, inexpensive, and timely index for the prevention and management of CAD.

## Data availability statement

The raw data supporting the conclusions of this article will be made available by the authors, without undue reservation.

## Ethics statement

The studies involving human participants were reviewed and approved by Ethics Review Committee of Qilu Hospital of Shandong University. The patients/participants provided their written informed consent to participate in this study. Written informed consent was obtained from the individual(s) for the publication of any potentially identifiable images or data included in this article.

## Author contributions

ZW, JY, and TZ drafted and revised the manuscript, contributed to the conception, and design of this manuscript. HC, WL, and YZ contributed to the case collection and database organization. LL, ZL, WZ, ZW, and JY performed the statistical analyses. All authors revised the manuscript critically for intellectual content, agreed to be accountable for the work and to ensure that any questions relating to the accuracy and integrity of the manuscript are investigated and properly resolved, and approved the final version.
