# Supplementary material for: Comparison of three non-insulin-based insulin resistance indexes in predicting the presence and severity of coronary artery disease
Source: Front Cardiovasc Med. 2022 Jul 29;9:918359. doi: 10.3389/fcvm.2022.918359 (PMC9374164; doi:10.3389/fcvm.2022.918359)
Supplement: Supplementary file 1 [file Data_Sheet_1.docx]

Supplementary Material

**Table S1.** Baseline characteristics of the study population according to the glucose metabolism status.

| **Variables** | **NGR** | **PDM** | **DM** | ***p*-value** |
| --- | --- | --- | --- | --- |
| **General conditions** |  |  |  |  |
| Age (years) | 58.55 ± 10.47 | 60.06 ± 9.24 | 59.27 ± 9.94 | 0.335 |
| Male, n (%) | 325 (67.1) | 69 (65.7) | 140 (65.7) | 0.916 |
| BMI (kg/m^2^) | 25.71 ± 3.35 | 26.96 ± 3.18 | 26.33 ± 2.99 | **0.001** |
| SBP (mmHg) | 133.04 ± 17.78 | 133.61 ± 16.62 | 134.62 ± 18.58 | 0.556 |
| DBP (mmHg) | 76.07 ± 11.06 | 75.01 ± 11.12 | 75.79 ± 11.15 | 0.500 |
| LVEF (%) | 60.01 ± 9.82 | 59.65 ± 9.13 | 58.86 ± 11.03 | 0.385 |
| GS | 30.50 (10.25-58.00) | 42.00 (17.50-77.50) | 48.00 (25.50-88.50) | **< 0.001** |
| CAD, n (%) | 368 (76.0) | 93 (88.6) | 192 (90.1) | **< 0.001** |
| **Risk factors, n (%)** |  |  |  |  |
| Current Smoking | 55 (11.4) | 6 (5.7) | 19 (8.9) | 0.180 |
| FH-CAD | 135 (27.9) | 27 (25.7) | 72 (33.8) | 0.202 |
| Hypertension | 275 (56.8) | 71 (67.6) | 148 (69.5) | **0.003** |
| **Laboratory test** |  |  |  |  |
| FPG (mg/dl) | 85.69 (79.47 – 91.68) | 104.52 (100.37 – 110.64) | 123.08 (99.92 – 149.84) | **< 0.001** |
| TC (mg/dl) | 148.07 (129.12 – 173.97) | 152.71 (129.90 – 178.61) | 148.98 (122.55 – 173.20) | 0.266 |
| TG (mg/dl) | 108.91 (84.34 – 142.34) | 131.93 (107.58 – 177.53) | 122.19 (90.32 – 158.94) | **< 0.001** |
| LDL-C (mg/dl) | 88.72 (71.62 – 110.08) | 95.49 (76.93 – 110.95) | 87.37 (70.17 – 112.69) | 0.136 |
| HDL-C (mg/dl) | 45.62 (39.82 – 52.96) | 44.46 (38.66 – 49.48) | 43.30 (37.89 – 50.06) | **0.004** |
| eGFR (ml/min/1.73m^2^) | 103.58 (91.20 – 117.88) | 105.31 (94.70 – 118.42) | 104.85 (93.35 – 122.10) | 0.305 |
| UA (μmol/L) | 305.00 (261.25 – 355.75) | 329.00 (270.00 – 388.50) | 289.00 (243.00 – 355.00) | **0.002** |
| **Cardiovascular medications, n (%)** |  |  |  |  |
| Single antiplatelet therapy | 86 (17.8) | 14 (13.3) | 52 (24.4) | **0.034** |
| Dual antiplatelet therapy | 10 (2.1) | 6 (5.7) | 4 (1.9) | 0.075 |
| Beta-blocker | 26 (5.4) | 12 (11.4) | 14 (6.6) | 0.073 |
| ACEI/ARB | 39 (8.1) | 7 (6.7) | 27 (12.7) | 0.096 |
| Statin | 81 (16.7) | 15 (14.3) | 39 (18.3) | 0.663 |
| **Diabetic medications, n (%)** |  |  |  |  |
| Insulin | 0 | 0 | 109 (51.2) | **< 0.001** |
| Metformin | 0 | 0 | 76 (35.7) | **< 0.001** |
| Other hypoglycemic drugs | 0 | 0 | 110 (51.6) | **< 0.001** |
| **TG/HDL-C ratio** | 2.38 (1.78 – 3.38) | 3.13 (2.27 – 4.53) | 2.87 (2.04 – 3.87) | **< 0.001** |
| **TyG index** | 8.43 (8.17 – 8.74) | 8.86 (8.59 – 9.20) | 8.91 (8.60 – 9.28) | **< 0.001** |
| **METS-IR** | 37.59 (33.90 – 42.05) | 42.15 (37.28 – 45.45) | 41.47 (36.74 – 45.50) | **< 0.001** |

*p*-values in bold are < 0.05.

NGR, normal glucose regulation; PDM, prediabetes mellitus; DM, diabetes mellitus; BMI, body mass index; SBP, systolic blood pressure; DBP, diastolic blood pressure; LVEF, left ventricular ejection fraction; GS, Gensini score; CAD, coronary artery disease; FH-CAD, family history of coronary artery disease; FPG, fasting plasma glucose; TC, total cholesterol; TG, triglyceride; LDL-C, low-density lipoprotein-cholesterol; HDL-C, high-density lipoprotein-cholesterol; eGFR, estimated glomerular filtration rate; UA, uric acid; ACEI, angiotensin-converting enzyme inhibitors; ARB, angiotensin receptor blockers; TG/HDL-C ratio, the ratio of triglycerides to high-density lipoprotein cholesterol; TyG index, triglyceride and glucose index; METS-IR, metabolic score for insulin resistance.

**Table S2.** Baseline characteristics of the patients with DM according to the use of metformin.

| **Variables** | **DM patients without metformin** | **DM patients with metformin** | ***p*-value** |
| --- | --- | --- | --- |
| **General conditions** |  |  |  |
| Age (years) | 58.64 ± 10.13 | 60.41 ± 9.55 | 0.213 |
| Male, n (%) | 100 (73.0) | 40 (52.6) | **0.003** |
| BMI (kg/m^2^) | 26.53 ± 2.86 | 25.96 ± 3.20 | 0.180 |
| SBP (mmHg) | 135.26 ± 18.11 | 133.47 ± 19.49 | 0.502 |
| DBP (mmHg) | 75.60 ± 11.90 | 73.96 ± 9.52 | 0.274 |
| LVEF (%) | 57.09 ± 11.76 | 59.85 ± 10.52 | 0.091 |
| GS | 58.00 (27.50-99.00) | 37.50 (23.25-76.50) | **0.015** |
| CAD, n (%) | 125 (91.2) | 67 (88.2) | 0.470 |
| **Risk factors, n (%)** |  |  |  |
| Current Smoking | 8 (5.8) | 11 (14.5) | **0.034** |
| FH-CAD | 49 (35.8) | 23 (30.3) | 0.416 |
| Hypertension | 96 (70.1) | 52 (68.4) | 0.802 |
| **Laboratory test** |  |  |  |
| FPG (mg/dl) | 124.16 (99.38 – 149.48) | 118.03 (104.61 – 155.20) | 0.730 |
| TC (mg/dl) | 143.82 (121.78 – 173.20) | 147.49 (122.64 – 174.45) | 0.774 |
| TG (mg/dl) | 115.99 (87.66 – 154.07) | 138.13 (95.19 – 178.63) | **0.045** |
| LDL-C (mg/dl) | 86.99 (69.39 – 112.69) | 88.53 (70.46 – 112.79) | 0.601 |
| HDL-C (mg/dl) | 42.14 (37.69 – 47.94) | 44.85 (38.85 – 51.90) | 0.053 |
| eGFR (ml/min/1.73m^2^) | 104.41 (91.65 – 121.53) | 105.11 (98.07 – 123.60) | 0.352 |
| UA (μmol/L) | 299.00 (254.00 – 357.00) | 279.00 (230.00 – 332.00) | **0.048** |
| **Cardiovascular medications, n (%)** |  |  |  |
| Single antiplatelet therapy | 19 (13.9) | 33 (43.4) | **< 0.001** |
| Dual antiplatelet therapy | 3 (2.2) | 1 (1.3) | 0.653 |
| Beta-blocker | 5 (3.6) | 9 (11.8) | **0.021** |
| ACEI/ARB | 16 (11.7) | 11 (14.5) | 0.557 |
| Statin | 15 (10.9) | 24 (31.6) | **< 0.001** |
| **TG/HDL-C ratio** | 2.81 (2.02 – 3.82) | 3.06 (2.06 – 3.95) | 0.383 |
| **TyG index** | 8.86 (8.54 – 9.25) | 9.01 (8.69 – 9.37) | 0.105 |
| **METS-IR** | 42.23 (36.70 – 46.41) | 40.41 (36.86 – 44.55) | 0.161 |

*p-*values in bold are < 0.05.

BMI, body mass index; SBP, systolic blood pressure; DBP, diastolic blood pressure; LVEF, left ventricular ejection fraction; GS, Gensini score; CAD, coronary artery disease; FH-CAD, family history of coronary artery disease; FPG, fasting plasma glucose; TC, total cholesterol; TG, triglyceride; LDL-C, low-density lipoprotein-cholesterol; HDL-C, high-density lipoprotein-cholesterol; eGFR, estimated glomerular filtration rate; UA, uric acid; ACEI, angiotensin-converting enzyme inhibitors; ARB, angiotensin receptor blockers; TG/HDL-C ratio, the ratio of triglycerides to high-density lipoprotein cholesterol; TyG index, triglyceride and glucose index; METS-IR, metabolic score for insulin resistance.
